# Evaluation of reliability and reproducibility of artificial intelligence-based, computer-based and traditional manual tracing methods

**DOI:** 10.1590/2177-6709.30.3.e2524157.oar

**Published:** 2025-10-13

**Authors:** Emel ARSLAN, Tugce SIRIN, Husamettin OKTAY, Delal Dara KILINC

**Affiliations:** 1 Istanbul Medipol University, Graduate School of Health Sciences, Department of Orthodontics (Istanbul, Turkey).; 2 Istanbul Medipol University, School of Dentistry, Department of Orthodontics (Istanbul, Turkey).; 3 Private practice (Istanbul, Turkey).

**Keywords:** Artificial intelligence, Orthodontics, Cephalometry, Nemoceph, Webceph, Inteligência artificial, Ortodontia, Cefalometria, Nemoceph, Webceph

## Abstract

**Introduction:**

Technological developments affect every aspect of life and science, including cephalometric evaluation methods in orthodontics. Artificial-intelligence-supported mobile Internet devices provide doctors the convenience of performing necessary analyses for patients anytime and anywhere.

**Objective:**

This study aimed to compare the lateral cephalometric measurement methods of the Artificial Intelligence Tracking Method (AITM, WebCeph), Computer-Based Tracking Method (CBTM, NemoCeph), and Manual Tracking Method (MTM), to evaluate the reliability of medical technological approaches, compared to traditional methods.

**Methods:**

Two hundred fifty lateral cephalometric radiographs were evaluated using three different methods: WebCeph, NemoCeph, and manual tracing. For this purpose, ten angular (SNA, SNB, ANB, Go-Gn/SN, Occlusal Plane/SN, U1/SN, U1/NA, L1/Mandibular Plane, L1/NB, and Interincisal angle [U1/L1]) and five linear (U1/NA, L1/NB, Wits Appraisal, Ls/E Plane, and Li/E Plane) parameters were measured, and the data were statistically analyzed.

**Results:**

There was excellent reliability between the three methods in measurements of SNA, SNB, L1/NB Angle, L1/NB mm, ANB, Wits Appraisal, Ls/E Plane, Li/E Plane, Go-Gn/SN, and Occlusal Plane/SN (p<0.001 and ICC range 0.915 to 0.979). Regarding the agreement between the two measurements, there were statistically significant perfect agreements in all other parameters, except for two moderate ones. Statistically significant differences were found in almost all measured parameters between the tracing methods.

**Conclusion:**

There were significant differences in the majority of the measurement methods. Regarding reproducibility, the Interincisal Angle values showed low consistency with the AITM method, and the U1/SN values showed low consistency with both the CBTM and AITM methods.

## RESUMO

**Introdução:**

Os avanços tecnológicos afetam todos os aspectos da vida e da ciência, inclusive os métodos de avaliação cefalométrica em Ortodontia. Aparelhos portáteis baseados em inteligência artificial oferecem aos dentistas a conveniência de analisar seus pacientes a qualquer hora e em qualquer lugar.

**Objetivo:**

O objetivo desse estudo foi comparar os métodos de análise cefalométrica de radiografias laterais por Rastreamento com Inteligência Artificial (Artificial Intelligence Tracking Method [AITM], WebCeph), por Rastreamento Baseado em Computador (Computer-Based Tracking Method [CBTM], NemoCeph) e do por Rastreamento Manual (Manual Tracking Method [MTM]), para avaliar a confiabilidade dessas abordagens tecnológicas, em comparação com os métodos tradicionais.

**Métodos:**

Duzentas e cinquenta radiografias cefalométricas laterais foram avaliadas usando os três métodos diferentes: WebCeph, NemoCeph e traçado manual. Para isso, foram medidos dez parâmetros angulares (SNA, SNB, ANB, Go-Gn/SN, Plano Oclusal/SN, U1/SN, U1/NA, L1/Plano Mandibular, L1/NB e Ângulo Interincisal [U1/L1]) e cinco lineares (U1/NA, L1/NB, Avaliação de Wits, Ls/Plano E e Li/Plano E), e os dados foram analisados estatisticamente.

**Resultados:**

Houve excelente confiabilidade entre os três métodos nas medições para SNA, SNB, ângulo L1.NB, L1/NB mm, ANB, avaliação de Wits, Ls/plano E, Li/plano E, Go-Gn/SN e plano oclusal/SN (p<0,001 e intervalo ICC de 0,915 a 0,979). Com relação à concordância entre as duas medições, houve concordância perfeita e estatisticamente significativa em todos os parâmetros, com exceção de dois moderados. Foram encontradas diferenças estatisticamente significativas entre os métodos de rastreamento em quase todos os parâmetros medidos.

**Conclusão:**

Houve diferenças significativas entre os métodos de medição na maioria das medições. Com relação à reprodutibilidade, os valores do ângulo interincisal apresentaram baixa consistência com o método AITM, e os valores de U1/SN apresentaram baixa consistência com os métodos CBTM e AITM.

## INTRODUCTION

Cephalometric analyses help to evaluate the dentofacial ratios of subjects, determine the anatomical basis of malocclusions, and analyze growth- and treatment-related changes. During orthodontic and orthognathic treatment planning, cephalometric analysis should be considered as a basic diagnostic method, especially when skeletal disharmony is suspected.^1^

Traditionally, cephalometric analyses are performed manually. These techniques, however, are time-consuming and may be affected by magnification in the cephalometric films.^2^ Despite its widespread use in Orthodontics, inaccuracies in identifying and measuring landmarks are a major concern.^3^ Deep learning has emerged as a revolutionary technical advancement in modern Orthodontics, offering novel methods for diagnosis, treatment planning, and outcome prediction. A common application of deep learning in contemporary Orthodontics is the automation of cephalometric analysis. To reduce human error and simplify the analysis process, deep learning algorithms are being trained to recognize anatomical features in radiographs.^4^ As a result of technological development, cephalometric measurements are being performed digitally, and significant improvements are achieved in terms of speed, quality, and reliability.^5^ Switching from the manual cephalometric analysis techniques to the digital ones provides many advantages to clinicians, but it requires professional supervision.^6^

Recently, smartphone applications have been developed for cephalometric analysis. In these applications, the operator manually defines the cephalometric reference points on the phone screen and then automatically calculates and displays the desired angular or linear measurements. These methods can be considered semiautomatic cephalometric analyses. Furthermore, some applications using artificial intelligence (AI) can automatically identify anatomical and cephalometric reference points and perform measurements when a digital cephalometric radiograph is loaded.^7^

AI is a transformative power in today’s digital revolution, affecting various economic sectors by performing tasks that typically require human intelligence.^8^ In the near future, web-based and AI-supported computer programs and smartphone cephalometric applications will be indispensable in orthodontic diagnosis and treatment planning, as they have multiple advantages such as time savings, when compared to manual measurements. However, time is not the only important factor. It is also very important that these new methods accurately determine anatomical and cephalometric landmarks and provide reliable and valid results.^9^

A smartphone application is a small-scale custom software program that can be downloaded onto a mobile device. The number of orthodontic apps in Google Play Store and Apple App Store has been increasing steadily.^10^ In 2020, automated web-based programs such as WebCeph have been introduced, which offer the possibility of automated landmark identification in lateral cephalometric radiographs.^11^ Such smart mobile technologies increase the possibilities of dental practice, and their consistent development has restructured the dental treatment process to include the use of mobile phone applications. Comparative studies are necessary because of the proliferation of smartphone applications, the availability of computer-aided cephalometric tracing programs, and the imprecision of commercially available software.^3^ Orthodontics uses computer technologies in diagnosis, treatment planning, and data storage more than other branches of Dentistry^12^ and has become a leading specialty in all fields of Dentistry.^13^

To the best of our knowledge, there are no published data comparing WebCeph (an AI-based smartphone application for cephalometric analysis), NemoCeph (a computer-based measurement method), and traditional manual measurement methods. Thus, the aim of the present study was to compare these cephalometric analysis software. The null hypothesis was that there would be no difference in reliability between them.

## MATERIALS AND METHODS

Ethics approval for this retrospective study was obtained from the university non-interventional clinical research ethics committee (number E-10840098-772.02-6758). Power analysis showed that a minimum sample size to be included in this study was 75 for each group with 95% confidence (1-α), 95% test power (1-β), and effect size of 0.47.^14^

The lateral cephalometric radiographs used in this study were randomly selected by examining the records of patients treated between 2020-2022. Although 225 cephalometric radiographs (75 for each group) would be sufficient, based on the results of the power analysis, 250 radiographs were selected from the archives and used in this study. The inclusion criteria for the radiographs were as follows: high-resolution images, full visibility of reference points, and a wide age range, regardless of sex and malocclusion type. Radiographs of patients with craniofacial anomalies or a history of surgical procedures were excluded. Poor image quality, artifacts on the images, studs/piercings, and the presence of any prosthesis, implants, and/or amalgam fillings were also exclusion criteria. All radiographs with calibration ruler images were obtained using the same machine and technique.

To standardize the study, the American Board of Orthodontics Cephalometric Monitoring References^15^ were used to select reference points and planes, and additional measurements were performed to enrich the data (Table 1). 

**Table 1:** Angular and linear parameters used in this study.

Angular parameters (degrees)	Linear parameters (mm)
SNA	U1/NA
SNB	L1/NB
ANB	Wits appraisal
Go-Gn/SN	Ls/E Plane
Occlusal plane/SN	Li/E Plane
U1/SN	
U1/NA	
L1/Mandibular plane	
L1/NB	
Interincisal angle (U1/L1)	

Although the analysis did not consider sex and age, the minimum and maximum ages of the participants and their average ages and sexes were recorded. As the values of the measurements in the present study were compared among the three methods, a classification was not made because the different dentofacial features of the individuals would not affect the results. Cephalometric radiographs were taken using the same X-ray machine (Kodak Trophy 9000C) by the same operator, with the patient in the natural head position, teeth at maximum closure, and lips closed without tension. 

Lateral radiographs were analyzed by three different methods: AI-based, computer-based, and manual measurements. For method error evaluation, 50 cephalometric radiographs were randomly selected and re-evaluated using the same three methods, two weeks after the completion of all measurements.

All radiographs were evaluated by a single researcher (EA). All measurements were performed by the same researcher (EA), to ensure consistency in the data evaluation and to ensure the reproducibility of the measurements.

### ARTIFICIAL INTELLIGENCE TRACING METHOD (AITM)

**Figure 1:**
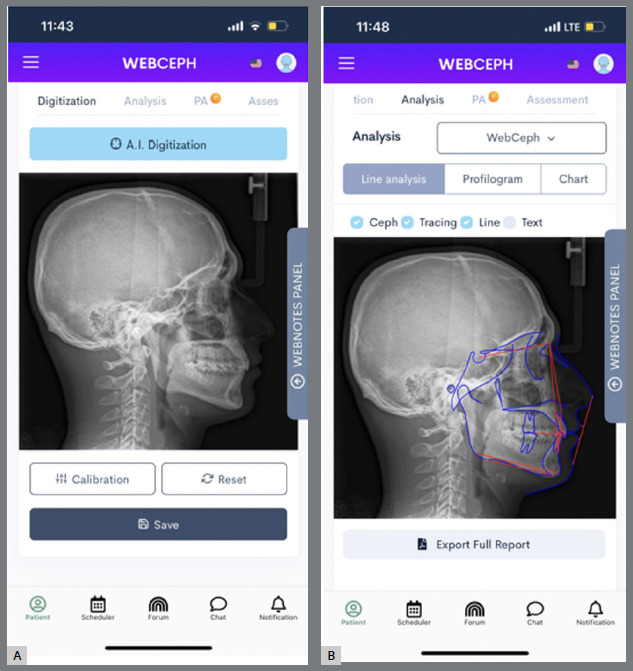
A) AITM, before landmark identification. B) AITM, after landmark identification.

High-quality JPEG images of the digital cephalograms (2.232 × 2.304 pixels, 150 dpi, and 8 bits) were downloaded to an iPhone 14 smartphone and transferred to the WebCeph application (WEBCEPH^TM^, Artificial Intelligence Orthodontic & Orthognathic Cloud Platform, South Korea, 2020) (Fig. 1A). Reference points were automatically defined and saved by the application, and the measurements listed in Table 1 were performed automatically (Fig. 1B).

### COMPUTER-BASED TRACING METHOD (CBTM)

**Figure 2:**
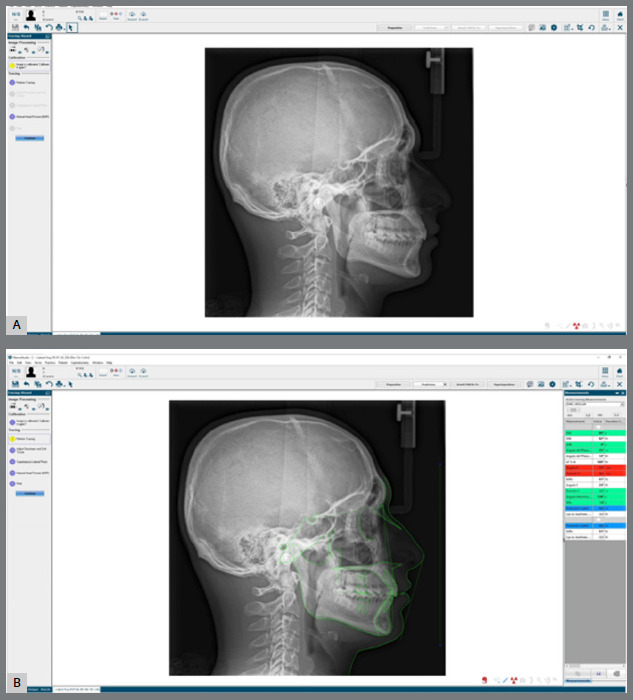
A) CBTM, before landmark identification. B) CBTM, after landmark identification ( by AI digitization ).

Digital radiographic images were saved in JPEG format for computer-based (Excalibur) measurements, and transferred to the NemoCeph program (Nemotec, Fall Edition 2020, Madrid, Spain) (Fig. 2A). The images were calibrated using a calibration ruler image on the radiographs, and the reference points of the cephalometric parameters were manually determined using a bookmark. Linear and angular measurements were performed automatically using the software (Fig. 2B).

### MANUAL TRACING METHOD (MTM)

**Figure 3:**
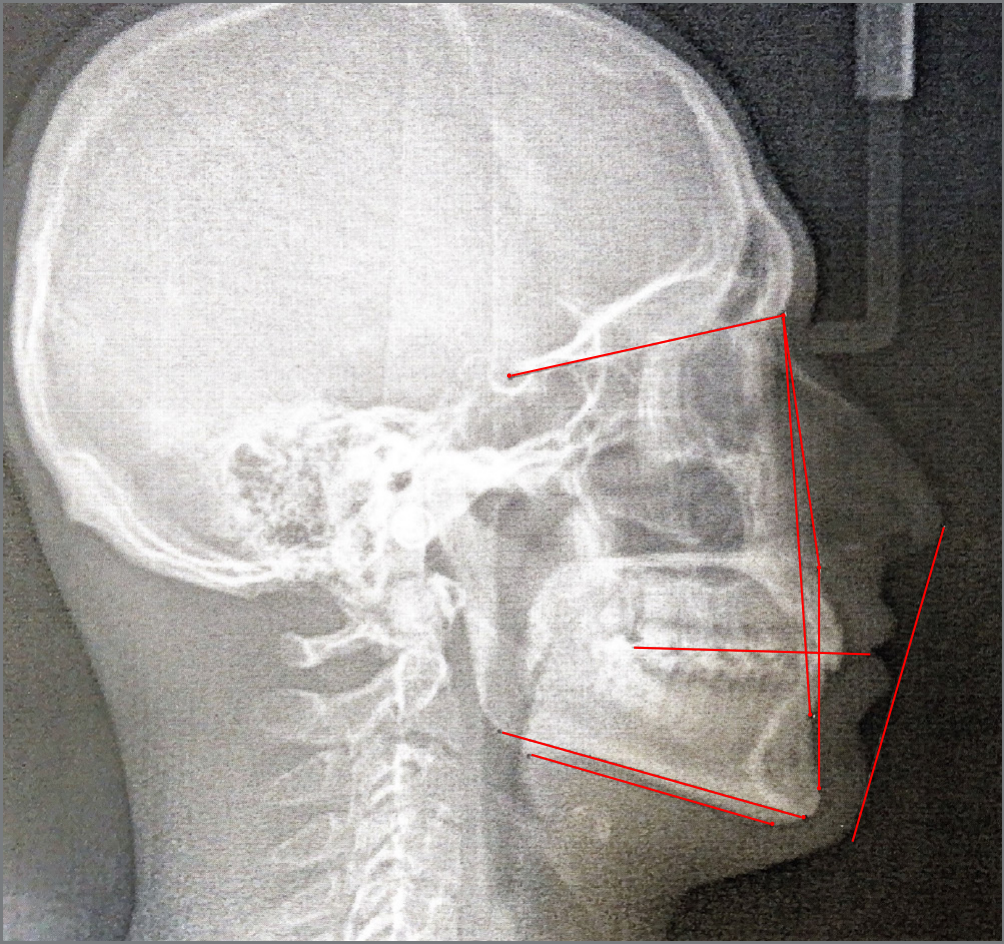
MTM, landmarks and measurements used in the study.

High-resolution digital cephalograms were printed on an A4 paper at a 1:1 scale. Cephalometric drawings were made using a 0.3-mm pencil. In cases with overlapping bilateral anatomical structures and/or double images, the midpoint of the two images was chosen as the reference point (Fig. 3). Linear and angular measurements were performed using a diagnostic ruler-protractor (Dentaurum 004-367-00).

### STATISTICAL ANALYSIS

Conformity of the measurements to normal distribution was evaluated using the Kolmogorov-Smirnov test, and the data were found to be normally distributed. The means and standard deviations of the investigated parameters were calculated. Between-group comparisons were made using analysis of variance (ANOVA) and multiple comparisons with Dunn’s test. To evaluate the method error, a paired sample t-test and intraclass correlation coefficient (ICC) were used. The significance level and confidence interval for all tests were set at p < 0.05 and 0.95, respectively. All statistical analyses were performed using the SPSS software package (IBM SPSS Statistics for Windows, version 23.0. Armonk, NY: IBM Corp.). 

To determine errors associated with digitization and measurement, 50 cephalometric radiographs were randomly selected. All procedures, such as landmark identification, tracing, and measurements, were repeated two weeks later by the same researcher. ICCs were calculated to assess the reliability of the measurements, using a paired t-test.

## RESULTS

Of the patients enrolled in the present study, 147 subjects (58.8%) were male, with a mean age of 17.79±7.01 years, and age range of 7.32 to 40.02 years; and 103 were female (41.2%) with a mean age of 16.50±6.98 years, and age range of 7.71 to 41.33 years. Mean age and range of total sample were 17.25 ± 7.02 years and 7.32 to 41.33 years, respectively.

### INTER-METHOD VALIDITY

There was an excellent, statistically significant agreement between the methods in terms of SNA (ICC=0.946; p<0.001); SNB (ICC=0.966; p<0.001); L1/NB Angle (ICC=0.958; p<0.001); L1/NB mm (ICC=0.965; p<0.001); ANB (ICC=0.952; p<0.001); Wits appraisal (ICC=0.94; p<0.001); Ls/E Plane (ICC=0.979; p<0.001); Li/E Plane (ICC=0.969; p<0.001); Go-Gn/SN (ICC=0.915; p<0.001) and Occlusal Plane/SN (ICC=0.949; p<0.001). There was a statistically significant moderate agreement between the methods in terms of U1/SN (ICC=0.735; p<0.001) and U1/NA angles (ICC=0.607; p<0.001). There was a statistically significant good agreement between the methods in terms of U1/NA mm (ICC=0.895; p<0.001); L1/Mandibular Plane (ICC=0.888; p<0.001), and Interincisal Angle (ICC=0.799; p<0.001) (Table 2).

**Table 2:** Correlation coefficient values of parameters between three different evaluation methods.

Parameters	ICC	95% CI of ICC
SNA	0.946	0.933 - 0.956
SNB	0.966	0.958 - 0.973
ANB	0.952	0.941 - 0.962
Go-Gn/SN	0.915	0.895 - 0.932
Occlusal Plane/SN	0.949	0.938 - 0.959
U1/SN	0.735	0.673 - 0.787
U1/NA Angle	0.607	0.514 - 0.684
U1/NA mm	0.895	0.87 - 0.916
L1/Mandibular Plane	0.888	0.862 - 0.910
L1/NB Angle	0.958	0.948 - 0.966
L1/NB mm	0.965	0.956 - 0.972
Interincisal Angle	0.799	0.752 - 0.839
Wits Appraisal	0.942	0.925 - 0.951
Ls/E Plane	0.979	0.974 - 0.983
Li/E Plane	0.969	0.962 - 0.975

*ICC (95% CI) = Interclass correlation coefficient (95% confidence interval).

### INTRA-RESEARCHER CONSISTENCY

The difference between the two readings made by the same researcher was tested to estimate the systemic error, using a paired t-test, which showed that there were no significant differences between the first and second examinations, confirming that all measurements were free from systemic errors (p>0.05). When examining the agreement between the researcher’s first and second measurements regarding the method error evaluation, there was a statistically significant moderate agreement between the two parameters, whereas there was a statistically significant perfect agreement for all other parameters.

In terms of U1/SN, there was a statistically significant perfect agreement between the researcher’s first and second measurements using the MTM method (ICC=0.986; p<0.001). In terms of U1/SN, there was a statistically significant moderate agreement between the first and second measurements of the researcher using the CBTM method (ICC=0.587; p=0.001). In terms of U1/SN, there was a statistically significant moderate agreement between the researcher’s first and second measurements using the AITM method (ICC=0.538; p=0.004).

In terms of the Interincisal Angle, there was a statistically significant perfect agreement between the researcher’s first and second measurements using the MTM method (ICC=0.994; p<0.001). In terms of the Interincisal Angle, there was a statistically significant perfect agreement between the researcher’s first and second measurements using the CBTM method (ICC=0.98; p<0.001). In terms of the Interincisal Angle, there was a statistically significant moderate agreement between the researcher’s first and second measurements using the AITM method (ICC=0.515; p=0.006) (Table 3).

**Table 3:** Evaluation of intra-examiner consistency between the researcher’s first and second measurements.

		ICC (%95 CI)	p
SNA	MTM	0.956 (0.922 - 0.975)	<0.001
	CBTM	0.943 (0.899 - 0.968)	<0.001
	AITM	0.992 (0.987 - 0.996)	<0.001
SNB	MTM	0.975 (0.957 - 0.986)	<0.001
	CBTM	0.979 (0.963 - 0.988)	<0.001
	AITM	0.998 (0.996 - 0.999)	<0.001
ANB	MTM	0.893 (0.811 - 0.939)	<0.001
	CBTM	0.941 (0.897 - 0.967)	<0.001
	AITM	0.998 (0.997 - 0.999)	<0.001
Go-Gn/SN	MTM	0.981 (0.967 - 0.989)	<0.001
	CBTM	0.945 (0.902 - 0.969)	<0.001
	AITM	0.997 (0.995 - 0.999)	<0.001
Occlusal Plane/SN	MTM	0.955 (0.922 - 0.975)	<0.001
	CBTM	0.949 (0.909 - 0.971)	<0.001
	AITM	0.99 (0.982 - 0.994)	<0.001
U1/SN	MTM	0.986 (0.976 - 0.992)	<0.001
	CBTM	0.587 (0.273 - 0.766)	0.001
	AITM	0.538 (0.186 - 0.738)	0.004
U1/NA Angle	MTM	0.982 (0.968 - 0.99)	<0.001
	CBTM	0.94 (0.894 - 0.966)	<0.001
	AITM	0.987 (0.977 - 0.993)	<0.001
U1/NA mm	MTM	0.944 (0.901 - 0.968)	<0.001
	CBTM	0.862 (0.758 - 0.922)	<0.001
	AITM	0.967 (0.941 - 0.981)	<0.001
L1/Mandıbular Plane	MTM	0.99 (0.982 - 0.994)	<0.001
	CBTM	0.941 (0.896 - 0.966)	<0.001
	AITM	0.998 (0.996 - 0.999)	<0.001
L1/NB Angle	MTM	0.986 (0.975 - 0.992)	<0.001
	CBTM	0.98 (0.965 - 0.989)	<0.001
	AITM	0.997 (0.995 - 0.998)	<0.001
L1/NB mm	MTM	0.973 (0.952 - 0.984)	<0.001
	CBTM	0.979 (0.963 - 0.988)	<0.001
	AITM	0.994 (0.989 - 0.997)	<0.001
İnterıncısor Angle	MTM	0.994 (0.99 - 0.997)	<0.001
	CBTM	0.98 (0.965 - 0.989)	<0.001
	AITM	0.515 (0.145 - 0.725)	0.006
Wits Appraisal	MTM	0.971 (0.949 - 0.983)	<0.001
	CBTM	0.942 (0.898 - 0.967)	<0.001
	AITM	0.994 (0.99 - 0.997)	<0.001
Ls/E Plane	MTM	0.981 (0.966 - 0.989)	<0.001
	CBTM	0.99 (0.983 - 0.994)	<0.001
	AITM	0.999 (0.998 - 0.999)	<0.001
Li/E Plane	MTM	0.983 (0.97 - 0.99)	<0.001
	CBTM	0.975 (0.955 - 0.986)	<0.001
	AITM	0.997 (0.994 - 0.998)	<0.001

*ICC (95% CI): Intraclass correlation coefficient (95% Confidence interval).

### CEPHALOMETRIC VALUES OBTAINED BY DIFFERENT METHODS

The mean and standard deviation values of the cephalometric parameters measured using the three different methods and the results of the comparison between the groups are presented in Table 4. The letters next to the mean values show the results of the Dunn test, which is a *post-hoc* test. The same letters in each row indicate that there was no statistically significant difference between the methods, whereas different letters indicate significant differences. As can be seen from the data in Table 4, statistically significant differences were found in nearly all parameters measured using the three tracing methods. In the manual tracing method, of the 15 parameters, L1/NB angular and linear measurements and the measurements of GoGn/SN and Ls/E planes did not show a difference from those of computer-based and AI tracing methods, respectively. Similarly, SNA, Occlusal Plane/SN, U1/NA mm, and Li/E Plane measurements showed no significant difference between the computer-based and AI tracing methods. Statistically significant differences were observed between the SNB, ANB, U1/SN, U1/NA, L1/Mandibular Plane, incisal angle, and Wits values according to the method (p<0.001) (Table 4).

**Table 4:** Mean and standard deviation and median values of parameters measured by three different methods and comparison results.

Parameters	Manual tracing	Computer-based tracing	Artificial intelligence tracing	p
SNA (Mean±SD)	81.30 ± 3.72	82.14 ± 3.67	81.85 ± 3.36	<0.001
SNA (Median [min-max])	81.00 (69.00 - 91.00)^a^	82.00 (71.00 - 92.00)^b^	82.05 (71.80 - 90.00)^b^	
SNB (Mean±SD)	78.35 ± 4.07	78.70 ± 4.22	77.48 ± 3.91	<0.001
SNB (Median [min-max])	78.00 (65.00 - 89.00)^a^	79.00 (65.00 - 91.00)^b^	77.30 (61.60 - 88.90)^c^	
ANB (Mean±SD)	2.96 ± 2.72	3.50 ± 2.72	4.37 ± 2.90	<0.001
ANB (Median [min-max])	3.00 (-6.00 - 8.00)^a^	4.00 (-6.00 - 9.00)^b^	5.00 (-5.60 - 13.90)^c^	
Go-Gn/SN (Mean±SD)	32.45 ± 7.74	31.28 ± 7.28	32.04 ± 6.57	<0.001
Go-Gn/SN (Median [min-max])	32.00 (8.00 - 94.00)^b^	31.00 (14.00 - 90.00)^a^	32.00 (8.90 - 53.70)^b^	
Occlusal Plane/SN (Mean±SD)	16.45 ± 4.70	16.91 ± 4.64	17.13 ± 4.82	<0.001
Occlusal Plane/SN (Median [min-max])	16.00 (5.00 - 36.00)^a^	17.00 (5.00 - 32.00)^b^	17.20 (4.70 - 37.00)^b^	
U1/SN (Mean±SD)	106.74 ± 13.69	106.37 ± 12.05	102.12 ± 10.02	<0.001
U1/SN (Median [min-max])	107.00 (2.00 - 203.00)^a^	106.50 (11.00 - 202.00)^b^	102.40 (11.70 - 131.50)^c^	
U1/NA Angle (Mean±SD)	26.85 ± 20.53	24.24 ± 8.27	20.74 ± 8.39	<0.001
U1/NA Angle (Median [min-max])	25.00 (2.00 - 322.00)^a^	24.00 (-3.00 - 47.00)^b^	20.95 (-5.70 - 46.00)^c^	
U1/NA mm (Mean±SD)	3.74 ± 2.69	3.30 ± 2.65	3.39 ± 2.30	<0.001
U1/NA mm (Median [min-max])	3.80 (-3.80 - 11.30)^a^	3.35 (-4.40 - 9.30)^b^	3.20 (-2.90 - 11.70)^b^	
L1/Mandibular Plane (Mean±SD)	94.15 ± 9.76	97.45 ± 8.69	95.64 ± 7.55	<0.001
L1/Mandibular Plane (Median [min-max])	95.00 (5.00 - 118.00)^a^	97.00 (69.00 - 122.00)^b^	95.35 (68.90 - 116.00)^c^	
L1/NB Angle (Mean±SD)	25.50 ± 7.16	25.83 ± 7.80	26.56 ± 6.56	<0.001
L1/NB Angle (Median [min-max])	26.00 (0.00 - 42.00)^a^	26.00 (5.00 - 43.00)^a^	27.05 (7.00 - 58.80)^b^	
L1/NB mm (Mean±SD)	4.57 ± 2.47	4.41 ± 2.64	5.65 ± 2.73	<0.001
L1/NB mm (Median [min-max])	5.00 (-1.30 - 11.30)^b^	4.45 (-2.10 - 13.20)^b^	5.60 (-1.50 - 16.20)^a^	
Interincisor Angle (Mean±SD)	123.73 ± 16.77	126.39 ± 12.43	127.37 ± 15.86	<0.001
Interincisor Angle (Median [min-max])	125.00 (23.00 - 165.00)^a^	126.00 (98.00 - 161.00)^b^	127.25 (2.60 - 163.40)^c^	
Wits Appraisal (Mean±SD)	0.50 ± 3.52	-0.15 ± 3.85	1.33 ± 4.83	<0.001
Wits Appraisal (Median [min-max])	1.30 (-10.00 - 13.80)^a^	0.30 (-13.40 - 10.80)^b^	1.65 (-15.50 - 14.30)^c^	
Ls/E Plane (Mean±SD)	-3.99 ± 3.05	-4.25 ± 3.26	-3.89 ± 3.48	<0.001
Ls/E Plane (Median [min-max])	-3.80 (-13.80 - 6.30)^b^	-4.00 (-15.80 - 5.20)^a^	-3.90 (-14.70 - 5.90)^b^	
Li/E Plane (Mean±SD)	-1.84 ± 3.16	-2.59 ± 3.22	-2.53 ± 3.34	<0.001
Li/E Plane (Median [min-max])	-1.90 (-11.30 - 7.50)^a^	-2.45 (-12.50 - 7.90)^b^	-2.55 (-11.30 - 12.20)^b^	

*Friedman test ^a-c^: There is no difference between methods with the same letter, mean ± standard deviation, median (minimum - maximum).

## DISCUSSION

MTM has long been the only available method for cephalometric analysis.^12^ However, digital cephalometric methods are currently in use. The advantages of digital methods, such as time savings and ease of use, are highly appreciated by clinicians. Furthermore, digitized methods allow image enhancement through adjustments in the magnification, contrast, and brightness. It should be noted that while tracing cephalometrics, no zooming can be performed on an analog image. However, using the zoom capability of a digital image or increasing/decreasing the screen light makes the selection of landmarks more suitable for digital images. Recent studies evaluated smartphone applications in Orthodontics. Nevertheless, applications are predicted to play a much greater role in the diagnosis, treatment planning, and prognostication of orthodontic patients in the future.^6^ ^,^ ^7^ ^,^ ^9^ ^,^ ^16^ ^,^ ^17^

Given the large amount of standardized and longitudinal multimodal data available, the application of AI in Orthodontics is promising and versatile. Nordblom et al.^18^ suggested that careful application of AI and machine learning methods should be the standard in AI research in Orthodontics. Several studies have examined the reliability and reproducibility of commercially available digital cephalometric software. In addition, as technology continues to develop at a rapid pace, cephalometric applications created specifically for smartphones offer useful opportunities for clinical applications. Furthermore, AI has made great strides in supporting clinicians in both the diagnostic and therapeutic areas of healthcare. Artificial intelligence mimics the human brain using a learning model that takes advantage of the characteristics of the existing data set.^9^ However, despite all their advantages, it is important to evaluate the reliability and reproducibility of digital cephalometric methods. 

In a previous study, it was mentioned that the accuracy and reliability of the applied method in terms of pinpointing anatomic cephalometric landmarks is more important than the applied method itself.^19^ Therefore, in the present study, the reliability and reproducibility of the fully automatic AI tracing method, semi-automatic computer-based tracing method, and manual cephalometric tracing methods were compared.

Paixão et al.^12^ compared manual and digital cephalometric tracings using Dolphin Imaging software with lateral radiograph measurements, and found consistency in all angular and linear measurements. Ahmed et al.^20^ did not find a statistically significant difference between manual tracing and WebCeph methods. Mitra et al.^21^ found no significant difference between manual, semi-automatic, and fully automatic digital cephalometric monitoring, with good agreement for all variables. It was stated that the preference to use a particular technique may depend on availability, expertise, and ease of maneuver. A higher ICC value (>0.9) was obtained for the following six parameters: ANB, L1-MP (degrees), LL-E-line, L1-NB (mm), L1-NB (degrees), S-N.Go-Gn. Five parameters - UL-E line, U1-NA (mm), SNA, SNB, U1-NA (degrees) - showed an ICC value between 0.75 and 0.90.^16^ In the study by Mahto et al.^17^ comparing fully automatic (WebCeph) and manual measurements, it was found an ICC value >0.75 in all measurements. In this study, statistically significant differences were found when the methods were compared, regarding SNB, ANB, U1−SN, U1−NA Angle, L1-Mandibular Plane, Interincisor Angle, and Wits appraisal values.

Kunz et al.^22^ showed significant differences between the estimates of four commercial providers (DentaliQ.ortho [CellmatiQ GmbH, Hamburg, Germany], WebCeph [AssembleCircle Corp, Seongnam-si, Korea], AudaxCeph [Audax d.o.o., Ljubljana, Slovenia], and CephX [Orca Dental AI, Herzliya, Israel]), and the human gold standard for all nine parameters was investigated. However, pairwise comparisons revealed significant differences between the four commercial providers. The authors found significant differences in the measurements of the SNA, SNB, ANB, L1/MeGn (p<0.01), and U1/SN (p<0.05), similar to the results of the present study.^22^

Sayar et al.^23^ compared the smartphone application (CephNinja) tracing and manual tracing methods. Similar to the present results, they found a statistically significant difference in SNB and ANB measurements. Zamrik et al.^24^ compared an Android cephalometric smartphone application (OneCeph) with the traditional method. Similar to the present results, they found a statistically significant difference in the SNB measurements. This could be due to the difficulty in pinpointing the exact location of the nasion when the nasofrontal suture was not clearly visualized. In addition, because point B lies on the curve, the measurement errors may be slightly higher.^25^

Sadek et al.^11^ conducted a study on 105 lateral cephalometrics to evaluate the accuracy of two web-based automatic cephalometric landmark identification and analysis programs. WebCeph (South Korea) and Cephio (Poland) were used for the automated cephalometric analysis. The authors concluded that most automated cephalometric measurements were clinically acceptable for most measurements.^11^

Kilinc et al.^9^ compared smartphone application tracing (CephNinja), web-based artificial intelligence tracing (WebCeph), and conventional hand-tracing methods. Similar to the present results, they found a statistically significant difference in the measurements of the U1/SN angle.^9^ Because the apical point of the upper incisor is difficult to see, it was thought that there could be a difference between the measurements of the U1/SN angle and the U1/NA angle.

Hassan et al.^26^ compared digital cephalometric monitoring using a smartphone application (app), tablet-based platform, and manual monitoring in thirty orthodontic patients. Thirty lateral cephalometric measurements were analyzed for Steiner analysis parameters (five skeletal, three dental, and one soft tissue) using three monitoring methods [manual - group GP M, smartphone (Android - OS9) - group GP S, and tablet (Apple, IOS13) - group GP T]. In contrast to the present study, the Steiner analysis parameters yielded similar results in all groups, with homogeneous variances. The differences between the measurements were not statistically significant (p<0.05). It was found that smartphone- and tablet-based apps produce comparable and reliable monitoring, compared to traditional manual monitoring.^26^

In contrast to the present study, Sayinsu et al.^27^ did not find a statistically significant difference between the measurements of the L1-MP angle.^27^ Since the upper incisors overlap with the lower incisors, it is more difficult to identify the incisal edges of the lower incisors than the upper incisors. It was thought that the difference in L1/Mandibular Plane angle measurements was due to this.

Duran et al.^28^ conducted a study to evaluate whether fully automated cephalometric analysis software with AI algorithms was as accurate as non-automated cephalometric analysis software for clinical diagnosis and research. The measurements in the study were made using the fully automatic digital cephalometric analysis software OrthoDx™ and WebCeph with an AI algorithm, and fully automatic markings, and measurements were made by the software. A weak level of consistency was found in linear measurement and soft tissue parameters in both software, and the difference between measurements was statistically found to be different from “0.” In parallel with the present study, the significance of the difference was at the 0.05 level.^28^

In contrast to the present findings, Meriç et al.^29^ and Erkan et al.^30^ did not find a statistically significant difference between the measurements in the interincisal angle in their studies. When measuring the interincisal angle, this parameter was thought to cause a difference between the measurements, because it is difficult to see the root tip in the bone, and it is difficult to see the incisor edges of the lower and upper incisors with the same sensitivity.

Çelik et al.^31^ did not find a statistically significant difference between measurements of the Wits appraisal in their study. Their findings were in contrast to the present findings. Determining the molar contact points while drawing the occlusal plane might be difficult because of the superposition of the contralateral molars, which could have caused the difference between the three methods in calculating the Wits appraisal value.

Gupta et al.^3^ compared the accuracy and speed of cephalometric analysis using an AI web-based method and a smartphone app-based system, with manual cephalometric analysis as the reference standard. OneCeph application was used in the application-based tracking method by the smartphone application; and Webceph™, as an online platform-based digital cephalometric software. The authors stated that cephalometric analyses performed using AI-based web-based and smartphone-application-based systems consumed less time than manual monitoring. Similar to the present study, they found significant differences in the SNB, SN to GoGn, and UI to NA (degrees) measurements. However, they found insignificant differences in the SNA, LI to NB (degrees), LI to NB (mm), Occ-SN, and UL E-line measurements.^3^

Meriç et al.^29^ compared app-aided tracing, computerized tracing, and manual tracing and, similar to the present results, they did not find a statistically significant difference in Ls/E Plane and Li/E Plane measurements.^29^ There was no significant difference between Ls/E Plane and Li/E Plane measurements in the study of Mahto et al.^17^, which was similar to the present study. These results were consistent with the results of the present study.

Danisman^32^ used lateral cephalometry of 60 patients in her study and compared the measurements of 20 parameters using both the AI-based platform WebCeph® and Dolphin Imaging. Most measurements were found to be statistically similar between Webceph and Dolphin, except for the SN-GoGn and IMPA angular measurements, and ANS-Me linear measurements. Similar to the results of the present study, there were statistically significant differences in the SN-GoGn angle. Unlike the present study, there were no significant differences between the SNA, SNB, ANB, U1/SN, U1/Na (degrees), U1/Na (mm), L1/Nb, L1/Nb (degrees), interincisal angle, Ls/E Plane, and Li/E Plane.^32^

In the present study, in manual measurements, it was thought that the thickness of the pencil tip could also have affected the measurement results, as a 0.3-mm hard black-tipped pencil was used to perform the measurements. The researchers recorded the numbers as integers. However, in the AI and computer-based measurement methods, the determined figure was measured and recorded as a decimal, thus providing more precise results.

Threshold measurements for clinically significant cephalometric differences vary in the literature; however, a difference that is statistically significant, but <2 measurement units (millimeters or degrees) is considered to be within clinically acceptable limits.^33^ ^,^ ^34^ The findings of the present study indicate that differences between MTM, CBTM, and AITM in some parameters could be large enough to affect the clinical outcomes of clinicians. Another important point that the authors want to emphasize in this study is that even if AI is used for the evaluation, different results can be obtained when repeated.

Alqahtani^35^ compared two online cephalometric software packages (FACAD and CephX), and reported that the differences between the two digital tracing methods - except for two angular measurements (SNA and FMA), and one linear measurement (Pg to NB) - were not statistically significant. All parameters, except POG to NB, ranged from moderate to nearly perfect agreement (>0.90). The authors added that the results obtained using both FACAD and CephX were reproducible, and although significant differences were obtained for some values, the differences were not clinically significant.^35^

Livas et al.^36^ compared Viewbox, OneCeph, and CephNinja cephalometric software. The authors concluded that OneCeph was highly valid, compared with Viewbox, whereas CephNinja was the most reliable. The authors added that smartphone apps performed satisfactorily in terms of validity and reliability.^36^

Bao et al.^37^ compared the accuracy of automatic cephalometric landmark localization and measurements using AI and computer-aided manual analysis. They detected 19 landmarks and obtained 23 measurements using computer-aided manual analysis (Dolphin Imaging 11.9) and automatic AI-based analysis (Planmeca Romexis v. 6.2). Soft tissue landmarks (1.54 ± 0.85 mm) had the highest consistency, while dental landmarks (2.37 ± 1.55 mm) had the highest variability. In total, 15 of the 23 measurements were within 2 mm or 2° of the clinically acceptable accuracy. The consistency rates were within the 95% limit of agreement for all measurement parameters and were >90%. Automatic analysis software was found to be sufficiently effective and acceptable in clinical practice for cephalometric measurements.^37^

Although three different tracing methods were evaluated in this study with a sample size larger than the number determined by power analysis, new studies with larger samples always make new contributions to the literature. Despite its strengths, this study had several limitations. In this article, the results presented by a single researcher are discussed. Future studies on this subject by other researchers will enrich the current literature. Different and complex intraoral conditions, such as image overlapping of the right and left teeth and incisors, could make it difficult for AI to recognize the related landmarks correctly. A further limitation of digital application usage is that they receive updates occasionally, which may influence the results based on the updated versions. Manual tracing has limitations, such as modifications to the printout based on printer settings and printer vibration-induced errors during printing.

## CONCLUSIONS

Thus, the null hypothesis was rejected. There were statistically significant differences in the cephalometric parameters measured by the three different methods in this study. 

The tracing results revealed significant differences in most measurements. There were no differences in L1-NB (mm), occlusal plane to SN, interincisal angle, or lower lip protrusion values between the three methods. Although statistically significant differences were found between the measurements acquired using the three methods, we believe that there were no clinically significant differences between them.

Regarding reproducibility, the Interincisal Angle values showed low consistency with the AITM method, and the U1/SN values showed low consistency with both the CBTM and AITM methods.
